# Impact of occupational exposures on exacerbation of asthma: a population-based asthma cohort study

**DOI:** 10.1186/s12890-016-0306-1

**Published:** 2016-11-15

**Authors:** Jeong-Lim Kim, Paul K. Henneberger, Susanna Lohman, Anna-Carin Olin, Anna Dahlman-Höglund, Eva Andersson, Kjell Torén, Mathias Holm

**Affiliations:** 1 Section of Occupational and Environmental Medicine, Sahlgrenska Academy at University of Gothenburg, Box 414, S-405 30 Gothenburg, Sweden; 2 National Institute for Occupational Safety and Health, Centers for Disease Control and Prevention, Morgantown, WV USA

**Keywords:** Asthma, Exacerbation, Job exposure matrix, Occupational exposure, Work exacerbated asthma, Workplace

## Abstract

**Background:**

Asthma is a prevalent chronic disease and occupation contributes to approximately 15 % of cases among adults. However, there are still few studies on risk factors for work-exacerbated asthma. The current study investigated the association between asthma exacerbations and occupational exposures.

**Methods:**

The study comprised all currently working adults (*n* = 1356) who reported ever asthma in prior population-based cohorts. All subjects completed a questionnaire about exposures, occupations and exacerbations of asthma. Exposure to high and low molecular weight agents, irritating agents and asthmagens were classified using the asthma-specific job exposure matrix for northern Europe (N-JEM). Severe exacerbation of asthma was defined as sought emergency care at a hospital, admitted to a hospital overnight, or made an urgent visit to a primary care physician or district medical office due to breathing problems during the last 12 months. Moderate exacerbation was defined as both being not severe exacerbation and an additional visit to a primary care physician or district medical office, or had extra treatments with corticosteroid tablets. Mild exacerbation was defined as being neither severe nor moderate exacerbation, and increasing usage of inhaled corticosteroids.

Multiple logistic regression was applied to investigate the association between exacerbation of asthma and occupational exposures while adjusting for potential confounders.

**Results:**

Approximately 26 % of the working asthmatics reported exacerbation, and more than two-thirds of them had moderate or severe exacerbation. From 23 to 49 % of the asthmatics reported occupational exposure to a variety of different types of agents. Exposure to any gas, smoke or dust (OR 1.7[95 % CI 1.2–2.6]) was associated with severe exacerbation of asthma, as were organic dust (OR 1.7[1.2–2.5]), dampness and mold (OR 1.8[1.2–2.7]), cold conditions (OR 1.7[1.1–2.7]), and a physically strenuous job (OR 1.6[1.03–2.3]). Asthmagens and low molecular weight agents classified by the N-JEM were associated with mild exacerbation, with OR 1.6[1.1–2.5] and OR 2.2[1.1–4.4], respectively.

**Conclusions:**

Self-reported exposure to any gas, smoke or dust, organic dust, dampness and mold, cold conditions and physically strenuous work, and jobs handling low molecular weight agents were associated with exacerbation of asthma. Reduction of these occupational exposures may help to reduce exacerbation of asthma.

## Background

Asthma is a prevalent chronic disease and occupational contribution to asthma has been reported as approximately 15 % among adult population [1]. Asthma due to occupational exposures is mainly separated into occupational asthma (asthma caused by work) and work-exacerbated asthma (WEA). Recent reviews have estimated that occupational exposures are causing 18 % of all adult-onset asthma [2] and that work-exacerbated asthma occurs in 22 % of adults with asthma [3, 4]. Exposures to irritant gases, fumes, dusts, chemicals, abnormal temperatures, poor indoor air quality and physically strenuous work have all been associated with WEA [5–8]. In contrast to occupational asthma, however, there are still few studies on risk factors for work-exacerbated asthma. In most of these studies, the subjects were asked if their aggravated asthma symptoms were associated with their work. This kind of question can cause bias by giving some information on the aim of the study.

Those with WEA seem to have a more severe asthma than workers with occupational asthma [9]. One recent study regarding work-exacerbated asthma showed that approximately 30 % of participants reported severe exacerbation [10]. They found a 2.5 fold-significantly increased risk for being exposed to inorganic dust among men and a 2-fold increased risk for low molecular weight reactive agents among women.

In the current study, we aimed to investigate WEA in a large study population by separating questions about specific occupational exposures from those used to define asthma exacerbation in a large study population. Also, we aimed to investigate WEA in relation to both several items on self-reported occupational exposures and the Nordic asthma-specific job exposure matrix (JEM) called the N-JEM [11, 12].

## Methods

### Study design and study population

The present study is based on an asthma cohort called Asthma-X that was derived from four different Swedish population-based studies: ADONIX [13], ALL000 [14, 15], MAP [16] and the Gothenburg part from the RHINE study [17], and one case-control study called M10 [18].

In brief, the ADONIX study includes a general population of men and women aged 25 to 75 years that were investigated between year 2001 and 2003. In total, 2,200 subjects completed a questionnaire and were clinically examined. The ALL000 study was carried out in all schoolchildren aged 15 and living in a county of West Sweden in year 2000. A questionnaire including items regarding respiratory symptoms was completed by 10,837 subjects. The MAP study included a random sample of the general population (*n* = 15,813), aged 20 to 50 years in 1993. The subjects completed a questionnaire regarding respiratory symptoms and smoking, of which 5.3 % reported asthma. The RHINE study was a follow-up of the random population samples in the European Respiratory Health Survey in Sweden, Norway, Denmark, Iceland and Estonia. For the RHINE study, a questionnaire including a self-reported asthma question was completed in a population aged 30–54 years (*n* = 14,731) during the follow-up period 1999–2001. The M10 study was carried out with a case-control design in 1996 and which consists of 321 adult asthmatics and 1,459 controls who completed a comprehensive questionnaire that included items regarding respiratory symptoms and details on occupation.

**Fig. 1:**
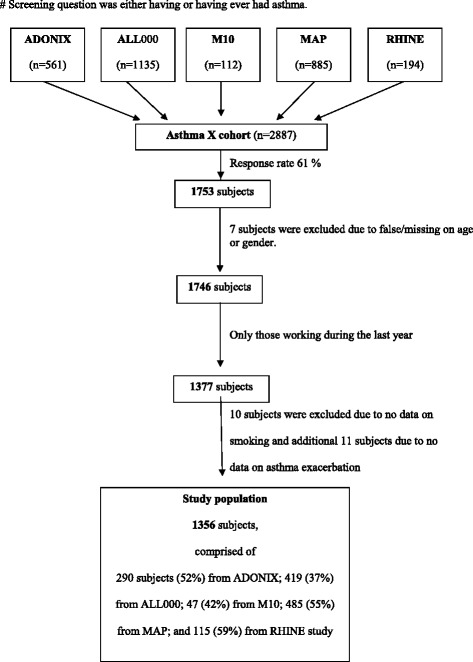
Flow diagram illustrating the study population

From these five previous studies, 2887 subjects who had reported that they have or ever had asthma were identified. In 2009, all of them were invited to participate in the current questionnaire-based survey and 1753 of them who agreed to participate completed the questionnaire and sent it back (response rate 61 %). Subjects with missing data on age or sex, or with a name that did not fully match were excluded (*n* = 7). We further restricted the study sample with complete data on subjects who had worked during the last year, smoking and exacerbation of asthma. This gave a final study population of 1356 subjects. Details of the study population are presented in Fig. 1.

### Questionnaire

The questionnaire comprised items about work and occupational exposure, acute breathing problems, respiratory problems, atopy and allergy, asthma medications, tobacco use and second-hand smoking (SHS), spare time and home environment.

Information on occupations and work tasks with at least 1 month of duration was requested, including the month and year of start and end for each occupation. We also asked about occupational exposures during the past 12 months, i.e., gas, smoke or dust; smell of frying; car exhaust fumes/engine fumes; mineral dust; organic dust (flour dust, wood dust, paper dust or textile dust); inorganic dust (grinding, milling, turning, mineral wool, glass wool or rock wool); welding or metal smoke; chemicals (strong acids, ammonia, formalin, cleaning chemicals, quick dry glue, cyanoacrylates, painting, lacquering or solvents); animals; dampness and mold (visible water damage, visible mold or smell of mold); cold (in cold store or outside during the winter); and physically strenuous job or frequent heavy lifting.

Second-hand smoke (SHS) was assessed by asking about regular exposure to other peoples’ tobacco smoke in the past 12-month period. *Atopy and allergy in childhood* were defined as an affirmative answer to the question, “Did you as a child have any form of allergy, for instance atopic dermatitis, asthma or hay fever?”

### Main outcome variables

The exacerbation variables were based on responses to questionnaire items that inquired about asthma-related care in the last 12 months. *Severe exacerbation of asthma* was defined as having sought emergency care at a hospital, admission to a hospital overnight, or having made an urgent visit to a primary care physician or district medical office due to breathing problems.

*Moderate exacerbation of asthma* was defined as both being *not severe exacerbation of asthma* and having made an additional visit to a primary care physician or district medical office, or taken any extra treatments with corticosteroids tablets due to breathing problems.

*Mild exacerbation of asthma* was defined as being not *severe* or *moderate exacerbation asthma*, and an affirmative answer to increasing usage of inhaled corticosteroids.

Any participant who fulfilled the criteria for one of the above-defined levels of exacerbation was considered as positive for “Overall exacerbation” and compared to those without exacerbation.

### Job Exposure Matrix (JEM)

The reported occupations were coded by an experienced occupational hygienist (ADH) according to the International Standard Classification of Occupations (ISCO-88) [19]. These codes were then linked to the newly developed N-JEM [11] (JEM for new onset of asthma in the countries in northern Europe) which included six main groups: high molecular weight agents (HMW); low molecular weight agents (LMW); irritating agents; accidental peak exposure to irritants; uncertain or low exposed group; and unexposed reference group. Moreover, we combined those who belonged to any of the following categories: HMW, LMW, irritating agents or accidental peak exposure into “Asthmagens” to investigate an overall exposure effect on respiratory health. In all the analyses, we used occupations held during the last 12 months. Subjects were allowed to belong to more than one JEM group if they had more than one occupation in the last year or more than one type of exposure in their job.

### Statistical methods

The continuous variables are presented as arithmetic mean with standard deviation (SD) and/or median with interquartile range (IQR), and categorical variables as frequency with percentages. Age was treated as a continuous variable in all regression models. Dummy variables were created for smoking status (never smoker versus ex-smoker and never smoker versus current smoker).

Chi-square test or Fisher’s exact test was performed to test differences in those with self-reported occupational exposures and in JEM groups with regard to exacerbation of asthma. To study associations between asthma exacerbation and potential predictors, multiple logistic regression models were performed with mutual adjustments for potential confounding factors. The reference group in each regression model was restricted to subjects who were not exposed to the specific occupational exposure, which means they might have had other types of work exposures. Multicollinearity was tested to detect the effect of inter-correlation among explanatory variables by using the variance inflation factor (VIF) and the results presented here were obtained by models with VIF values below 2. Hosmer–Lemeshow goodness-of-fit statistics were used to assess the calibration of the models, and all models which met the criteria (p > 0.05) are presented in either text or tables. The results of regression analyses are presented as odds ratios with 95 % confidence intervals (CI). All statistical analyses were performed using version 9.3 of SAS for Windows (SAS Institute, Inc., Cary, NC, USA), applying two-tailed tests and a 5 % level of significance.

## Results

Demographic characteristics of the study population are presented in Table 1. Approximately 47 % were men and the mean age was 43 years (SD 14.2) and the median was 44 (range 25–77 and IQR 25–55). Eleven per cent of the subjects were current smokers and a similar proportion had regularly been exposed to SHS. However, the rate of regular SHS at work was low (1.9 %). Onset of asthma after the age of 15 was reported by 21 % and current asthma, which is defined as either asthma symptoms or medication during the last 12 months, was reported by 52 %.

**Table 1:** Demographic characteristics of the study population and exacerbation of asthma

	Study population (*n* = 1356)
	N (%)
Sex	
Women	723 (53.3)
Men	633 (46.7)
Age	
Mean (standard deviation)	42.5 (14.2)
Median (interquartile range)	44.0 (25–55)
Smoking status	
Never smoker	794 (58.6)
Ex- smoker	411 (30.3)
Current smoker	151 (11.1)
Regular second hand smoke exposure in the past 12 months	150 (11.1)
Atopy and allergy in childhood	981 (72.9)
Onset of asthma after the age of 15	284 (20.9)
Exacerbation of asthma	
Mild	107 (8.1)
moderate	118 (8.7)
Severe	133 (9.8)

Mild exacerbation of asthma during the past 12 months was reported by 8.1 %, moderate exacerbation by 8.7 % and severe by 9.8 %. (Table 1). Severe exacerbation of asthma was more common among females (*p* < 0.01), current smokers (*p* < 0.05) and those exposed to SHS (*p* < 0.01) (data not shown). There was no such pattern regarding mild or moderate exacerbation of asthma.

Based on crude comparisons, both self-reported and N-JEM classified occupational exposures did not differ between those with overall exacerbation and without exacerbation (Table 2). By dividing into different definition categories of exacerbation, however, moderate exacerbation of asthma was more common in those with self-reported exposure to animals (only 5 % exposed, *p* < 0.05) and severe exacerbation was more common in those with self-reported exposures to any gas, smoke or dust (*p* < 0.05), organic dust (*p* < 0.01), dampness and mold (*p* < 0.01) and working in cold conditions (*p* < 0.05). These exposures were reported by a large proportion of the study population, ranging from 23 to 50 %.

**Table 2:** Self-reported work exposure during the past 12 months and Job Exposure matrix (JEM)

	Study population (*n* = 1356)	Asthma exacerbation							
		Mild (*n* = 107)		Moderate (*n* = 118)		Severe (*n* = 133)		Overall (*n* = 358)	
	N (%)	N (%)	*p*-value*	N (%)	*p*-value*	N (%)	*p*-value*	N (%)	*p*-value*
Self-reported work exposure in the past 12 months									
Gas, smoke or dust	593 (44.6)	52 (50.0)	0.22	48 (40.7)	0.49	69 (53.5)	0.03	169 (48.2)	0.15
Smell of frying	179 (13.7)	16 (15.8)	0.44	18 (15.4)	0.50	20 (15.6)	0.50	54 (15.6)	0.25
Car exhaust fumes/engine fumes	311 (23.7)	23 (23.0)	0.86	26 (22.2)	0.71	31 (24.2)	0.88	80 (23.2)	0.82
Mineral dust	154 (11.7)	12 (12.1)	0.99	14 (12.0)	0.98	13 (10.2)	0.57	39 (11.3)	0.71
Organic dust^a^	461 (34.9)	34 (34.0)	0.88	41 (35.3)	0.68	60 (46.5)	<0.01	135 (39.1)	0.05
Inorganic dust^b^	211 (16.0)	17 (16.7)	0.93	18 (15.4)	0.81	19 (15.0)	0.74	54 (15.6)	0.75
Welding or metal smoke	166 (12.6)	12 (11.9)	0.79	16 (13.6)	0.78	14 (11.1)	0.61	42 (12.2)	0.76
Chemicals^c^	512 (38.9)	46 (45.5)	0.17	46 (39.3)	0.88	52 (40.6)	0.67	144 (41.6)	0.28
Animals	157 (11.9)	16 (15.5)	0.28	6 (5.2)	0.02	19 (15.2)	0.24	41 (12.0)	0.97
Dampness and mold^d^	299 (22.6)	23 (22.3)	0.96	22 (18.8)	0.46	42 (32.3)	<0.01	87 (24.9)	0.30
Cold (in cold store/outside during the winter)	314 (23.7)	26 (25.2)	0.57	29 (25.0)	0.58	40 (30.8)	0.05	95 (27.2)	0.10
Physically strenuous job^e^	649 (48.8)	54 (52.4)	0.41	50 (42.4)	0.21	73 (56.6)	0.06	177 (50.6)	0.45
Job Exposure Matrix (JEM) by the job in the last 12 months									
Unexposed any of exposure below	704 (51.9)	50 (46.7)	0.21	59 (50.0)	0.63	66 (49.6)	0.58	175 (49.0)	0.17
Asthmagens^f^	530 (39.1)	50 (46.7)	0.07	48 (40.7)	0.69	54 (40.6)	0.71	152 (42.5)	0.13
High molecular weight agents (HMW)	235 (17.3)	25 (23.4)	0.09	20 (17.0)	0.91	23 (17.3)	0.99	68 (19.0)	0.34
Low molecular weight agents (LMW)	100 (7.4)	12 (11.2)	0.14	8 (6.8)	0.74	8 (6.0)	0.53	28 (7.8)	0.71
Irritating agents	286 (21.1)	22 (20.6)	0.99	29 (24.6)	0.32	30 (22.6)	0.66	81 (22.6)	0.41
Accidental peak exposure to irritants	15 (1.1)	3 (2.8)	N.A	1 (0.9)	N.A	2 (1.5)	N.A.	6 (1.7)	0.27
Uncertain/low exposed	132 (9.7)	7 (6.5)	0.26	11 (9.3)	0.89	14 (10.5)	0.75	32 (8.9)	0.58

^*^P-value was obtained by Chi-square test or Fisher’s exact test comparing differences in those with yes vs. no in different categories of exacerbation

^a^Flour/wood/paper/ textile dust

^b^Grinding/milling/turning/mineral wool/glass wool/rock wool

^c^Cleaning chemicals/ strong acids/ammonia/formalin/quick dry glue or cyanoacrylates/painting or lacquering/solvents

^d^Visible water damage, visible mold or smell of mold

^e^Physically strenusous job/frequent heavy lifting

^f^Any of exposure to HMW, LMW, Irritating agents or peak exposure to irritants

According to N-JEM, our study population was allocated into approximately 17 % exposed to HMW, 7 % LMW and 21 % irritating agents. (Table 2). From comparisons of the crude data, the prevalence of asthma exacerbation did not significantly differ by the different N-JEM categories. For further analyses in the current investigation, the category of accidental peak exposure to irritants was excluded due to too few subjects.

When performing multiple logistic regression, overall asthma exacerbation was associated with self-reported exposures to any gas, smoke or dust (OR 1.4, 95 % CI 1.1–1.8), organic dust (OR 1.4, 95 % CI 1.0–1.8) and working in cold conditions (OR 1.6, 95 % CI 1.2–2.2) (Table 3). When restricting analyses to severe exacerbation of asthma, these associations were even stronger with ORs of approximately 1.7. Moreover, severe exacerbation of asthma was more pronounced among those who reported a workplace with dampness and mold (OR 1.8, 95 % CI 1.2–2.7), and a physically strenuous job (OR 1.6, 95 % CI 1.0–2.3). None of these associations was found among those with mild or moderate exacerbation.

**Table 3:** Multiple logistic regression models for exacerbation of asthma in relation to self-reported work exposure in the last 12 months (*n* = 1356)

	Mild	Moderate	Severe	Overall
Predictor categories	OR (95 % CI)	OR (95 % CI)	OR (95 % CI)	OR (95 % CI)
Exposure in the past 12 months^a^				
Gas, smoke or dust	1.48 (0.96–2.28)	0.94 (0.62–1.42)	**1.74 (1.17–2.58)**	**1.39 (1.07–1.81)**
Smell of frying	1.33 (0.74–2.38)	1.38 (0.78–2.42)	0.92 (0.53–1.60)	1.20 (0.83–1.74)
Car exhaust fumes/engine fumes	1.03 (0.61–1.76)	1.09 (0.67–1.77)	1.12 (0.70–1.77)	1.10 (0.80–1.51)
Mineral dust	1.23 (0.63–2.42)	1.10 (0.58–2.10)	1.10 (0.58–2.08)	1.15 (0.76–1.74)
Organic dust	1.10 (0.71–1.71)	1.17 (0.77–1.77)	**1.72 (1.18–2.51)**	**1.36 (1.04–1.76)**
Inorganic dust	1.30 (0.71–2.39)	0.92 (0.51–1.68)	1.25 (0.70–2.21)	1.15 (0.79–1.67)
Welding or metal smoke	1.14 (0.58–2.25)	1.07 (0.57–2.00)	1.12 (0.59–2.12)	1.12 (0.74–1.68)
Chemicals	1.52 (0.99–2.34)	1.12 (0.74–1.70)	1.10 (0.74–1.63)	1.25 (0.96–1.62)
Animals	1.53 (0.84–2.78)	0.48 (0.20–1.13)	1.07 (0.61–1.90)	1.00 (0.67–1.50)
Dampness and mold	1.08 (0.66–1.78)	0.96 (0.59–1.57)	**1.79 (1.19–2.67)**	1.25 (0.93–1.68)
Cold (in cold store/outside during the winter)	1.48 (0.95–2.31)	1.44 (0.89–2.34)	**1.74 (1.12–2.69)**	**1.59 (1.17–2.15)**
Physically strenuous job	1.48 (0.95–2.31)	0.86 (0.56–1.31)	**1.55 (1.03–2.32)**	1.29 (0.99–1.69)

^a^Each model was separately performed for each type of self-reported work exposure adjusting for gender (reference = female), age (continuous variable), current smoker, Second-hand smoke (SHS) and history of self-reported allergy. The reference group in each model was the unexposed subjects for the specific occupational exposure category which means they might have had other types of work exposures. *P*-values <0.05 are marked bold

When all the self-reported occupational exposures associated with severe exacerbation in prior analyses were included in one regression model, severe exacerbation of asthma was still associated with exposure to organic dust (OR 1.5, 95 % CI 1.0–2.4) and to dampness and mold (OR 1.7, 95 % CI 1.1–2.6) but far from significant association to exposure to any gas, smoke or dust, working in cold conditions or physically strenuous job (p > 0.1) (data not shown). However, overall exacerbation of asthma was no longer significantly associated with any self-reported occupational exposures in analysis with all exposures in one model. When the model was stratified by sex, the main findings remained similar but the association between severe exacerbation of asthma and self-reported physically strenuous job was significant only in males (OR 1.9, 95 % CI 1.0–3.8) but not in females (OR 1.4, 95 % CI 0.8–2.3) (data not shown).

From multiple regression models with covariate for exposure categories, asthmagens (OR 1.6, 95 % CI 1.01–2.5) and LMW (OR 2.2, 95 % CI 1.1–4.4) classified by the N-JEM were associated with mild exacerbation of asthma, and asthmagens (OR 1.3, 95 % CI .1.01–1.7) was associated with overall exacerbation (Table 4). However, neither moderate nor severe exacerbation of asthma was associated with an exposure category by the N-JEM.

**Table 4:** Multiple logistic regression models for exacerbation of asthma in relation to JEM in the last 12 months^a^

	Mild	Moderate	Severe	Overall
Predictor categories	OR (95 % CI)	OR (95 % CI)	OR (95 % CI)	OR (95 % CI)
Unexposed (reference, *n* = 704)	1	1	1	1
Asthmagens (*n* = 530)^b^	**1.62 (1.06–2.49)**	1.17 (0.77–1.78)	1.07 (0.72–1.59)	**1.32 (1.01–1.72)**
High molecular weight agents (HMW, *n* = 235)	1.68 (0.98–2.88)	1.02 (0.58–1.82)	0.96 (0.57–1.62)	1.22 (0.86–1.74)
Low molecular weight agents (LMW, *n* = 100)	**2.16 (1.05–4.44)**	0.97 (0.42–2.26)	0.91 (0.39–2.12)	1.35 (0.82–2.25)
Irritating agents (*n* = 286)	1.29 (0.73–2.30)	1.36 (0.81–2.30)	1.25 (0.75–2.09)	1.36 (0.96–1.92)
Uncertain/low exposed (*n* = 132)	0.77 (0.33–1.76)	1.08 (0.54–2.14)	1.40 (0.75–2.63)	1.10 (0.70–1.71)

^a^Each model was separately performed for each type of work exposure adjusting for sex (reference = female), age (continuous variable), current smoker, second hand smoke (SHS) and atopy and allergy in childhood. *P*-values <0.05 are marked bold

^b^Any of exposure to HMW, LMW or Irritating agents

## Discussion

In the current cohort of working asthmatics, the prevalence of exacerbation of asthma during the last 12 months was approximately 26 % and more than two-thirds of them had moderate or severe exacerbation. The 9.8 % of current participants with severe exacerbation was only somewhat greater than the 7.7 % of working adults with asthma who fulfilled similar criteria for severe exacerbation in the European Community Respiratory Health Survey II [6]. A substantial proportion of the study population reported occupational exposures to chemicals, gas, smoke, dust, fumes, dampness and mold, and work in the cold conditions. Females and current smokers reported more exacerbations of asthma, which is in good agreement with previous findings [6, 20]. Those exposed to SHS were also more prone to report asthma exacerbations.

Self-reported exposure to any gas, smoke or dust, and organic dust, and working in cold conditions, were associated with overall exacerbation of asthma. Moreover, self-reported exposure to dampness and mold, and physically strenuous job were associated with severe exacerbation of asthma. Exposure to “asthmagens” and “low molecular weight agents” in the N-JEM were significantly associated with mild exacerbation of asthma but no significant association with the other categories in the N-JEM was found.

The strength of the current study of asthma exacerbation is the detailed questioning on work exposures regardless of any respiratory symptoms and recent job history in a large cohort of asthmatics. Moreover, the reported occupations were coded and linked to a newly developed asthma-specific JEM that was intended for use in northern Europe [11, 12]. Thus, both self-reported and JEM-assessed occupational exposures were analyzed in relation to exacerbations of asthma.

Our study also has some potential methodological limitations. The cross-sectional design of the study implies a risk of recall bias. In order to reduce this type of bias, we separated the exposure questions from those used to define asthma exacerbation. In order to establish an unbiased assessment of occupational exposure we also used the N-JEM. Another potential limitation is that exacerbation of asthma was defined by using a self-administered questionnaire although this approach is commonly used in epidemiological studies. However, the questions we used to define asthma exacerbation are distinct, and especially the questions used to define a severe exacerbation (achieving the highest risk-estimates), i.e., to seek acute help from a health care provider seem easy to remember and we think the answers to the questions match the real circumstances well.

Another possible limitation is that subjects in the current asthma cohort study were derived from five different studies and one of them was carried out among relatively young adults (15 years old in the year 2000) [14, 15]. The total response rate was approximately 61 % but in the young adult population study it was as low as 44 %, which may cause some under-representation of young asthmatics in the present study. Also, the low participation percentage among young adults might imply that selection bias may be more likely than among older participant groups. Nonetheless, there might be sufficient accumulation of young participants with asthma since a large proportion of the current asthma cohort was derived from the young population study ALL000. Since age distribution varies among the five studies, consequently, we controlled for potential confounding by this factor in all the models. Furthermore, we have studied subjects that reported “ever asthma” in previous surveys, of which a large proportion reported onset of asthma before the age of 16. Consequently, it follows that individuals with asthma in remission were included, and some participants may only have had transient asthma during childhood. This probably resulted in a lower prevalence of asthma exacerbation than if only subjects with an ongoing asthma had been studied.

The definition of *severe asthma exacerbation* has not been constant in the literature. However, in a recent American Thoracic Society (ATS)/European Respiratory Society (ERS) statement severe asthma exacerbations were defined as events that require use, or an increase from a stable dose, of systemic corticosteroids and/or hospitalization or emergency room visit [21] which is close to our definition. Less severe exacerbations seem more difficult to define and it is hard to draw a distinct line between moderate exacerbations and normal fluctuations in the asthma disease, especially in questionnaire-based studies. In the ATS/ERS consensus statement [21], a moderate asthma exacerbation was defined as an event that result in a temporary change in treatment that is intended to prevent the exacerbation from becoming severe. For the current study, we have a large number of events which allowed us to use the modified ATS/ERS consensus statement to three severity grades: Severe, Moderate and Mild; and overall asthma exacerbation regardless of severity grades. We also found the strongest associations for the severe exacerbations, where the definition is most precise.

So far, relatively few studies have attempted to demonstrate work-related risk factors for severe exacerbation of asthma or aggravated asthma symptoms [6, 8, 10]. An increased risk of severe exacerbation of asthma among those exposed to gas/fume, mineral dust or any dust at work has been shown [5–7]. A recent study showed that high exposure to gas, dust or fumes was significantly associated with severe exacerbation of asthma (relative risk 2.5 with 95 % CI 1.2–5.5) [6]. In the current study, we found similar results with a significant association between severe exacerbation of asthma and self-reported exposure to any gas, smoke or dust. Moreover, self-reported exposure to organic dust was associated with severe exacerbation of asthma. We also found that self-reported problems with dampness/mold at work were significantly associated with severe exacerbation of asthma, which is in good agreement with previous literature. Numerous studies have shown that exposure to dampness and biological contaminants in indoor environment can be adversely associated with asthma and respiratory symptoms [22, 23]. Self-reported work in cold conditions and physically strenuous job were both associated with asthma exacerbations, which is in line with results from a study by Saarinen et al. 2003 [8], where such exposures were associated with work-aggravated asthma symptoms. We found slightly different pictures of association between occupational exposure and exacerbation of asthma, when comparing self-reported and N-JEM categories. That is, the self-reported “any exposure to gas, smoke or dust” and “exposure to organic dust” are exposures that fit under the “irritating agents” and “asthmagens” N-JEM category. A possible explanation for the finding may be information bias. Previously, it was shown that the prevalence of self-reported occupational exposure depended on asthmatic health status [24]. In other words, subjects with severe asthma might be more likely to report their occupational exposure than others. The association of N-JEM “asthmagens” with mild exacerbation alone might indicate that the exposure was sufficient to result in this mild response but not moderate or severe exacerbation. It is also possible that some imprecision in the exposure metric compromised the ability to detect an association with the moderate or severe outcome. One has to bear in mind that the N-JEM is an asthma-specific JEM that was developed with special focus on detection of new-onset asthma rather than exacerbation of asthma. The vast majority of participants in the current study probably did not have occupational asthma, but rather asthma caused by factors outside their work. Apart from the fact that agents that are causing asthma do not necessarily trigger exacerbations of the disease, it can be that those who are already suffering from asthma are likely to develop exacerbations at lower levels of exposures than those levels needed to initiate new-onset asthma. In fact, Lemiere and co-authors concluded in a recent study [9] that subjects with work-exacerbated asthma appeared to have greater asthma severity than those with occupational asthma. In short, there is a possibility that both exposures and exposure levels of interest for WEA are not fully covered by the JEM we used, consistent with the fact that it is challenging to accurately assess occupational exposures in a questionnaire-based setting.

## Conclusion

Adults with asthma who reported occupational exposure to any gas, smoke or dust; organic dust; dampness and mold; cold conditions; or physically strenuous work, and who had jobs working with low molecular weight agents had increased risk of exacerbation of asthma. To diminish exacerbation of asthma, it may help to reduce such exposures in workplaces.

## Abbreviations

ATS: American thoracic society
CI: Confidence interval
ERS: European respiratory society
HMW: High molecular weight agents
IQR: Interquartile range
JEM: Job exposure matrix
LMW: Low molecular weight agents
N-JEM: The Nordic asthma-specific job exposure matrix
SD: Standard deviation
SHS: Second-hand smoking
VIF: Variance inflation factor
WEA: Work-exacerbated asthma
